# The Anti-Endometriotic Effect of Cyperi Rhizoma Extract, Inhibiting Cell Adhesion and the Expression of Pain-Related Factors through Akt and NF-kB Pathways

**DOI:** 10.3390/medicina58030335

**Published:** 2022-02-23

**Authors:** Ji-Hye Ahn, Jun-Myeong Choi, Eun-Sol Kang, Jae-Hyeon Yoo, Yoon-Jin Cho, Dae Sik Jang, Jung-Hye Choi

**Affiliations:** 1 Department of Oriental Pharmacy, Woosuk University, Jeonju 55338, Korea; 2 Department of Oriental Pharmaceutical Science, College of Pharmacy, Kyung Hee University, Seoul 02447, Korea; tkarjawhfh@gmail.com (J.-M.C.); amorfati8147@naver.com (E.-S.K.); gaaon@hanmail.net (J.-H.Y.); cyjin422@khu.ac.kr (Y.-J.C.); 3 Department of Biomedical and Pharmaceutical Sciences, Kyung Hee University, Seoul 02447, Korea; dsjang@khu.ac.kr

**Keywords:** Cyperi rhizome, endometriosis, adhesion, neurotrophins, Akt, NF-kB

## Abstract

Rhizomes of *Cyperus rotundus* have been widely used as a traditional medicine in Asia for the treatment of gynecological diseases. However, there is no scientific evidence demonstrating the effect of *C. rotundus* rhizomes on endometriosis, which is characterized by the adhesion of endometrial tissues outside the uterus, resulting in chronic and severe pelvic pain. The aim of this study was to investigate the effects of Cyperi rhizoma extract (CRE) on cell adhesion and the expression of pain-related factors (neurotrophins) in endometriotic cells, and to elucidate the underlying molecular mechanisms. CRE inhibited the adhesion of human endometriotic 12Z cells to peritoneal mesothelial Met5A cells using by adhesion assays. The mRNA expression of adhesion molecules [P-cadherin and matrix metalloproteinase (MMP)-2] was downregulated by CRE treatment. In addition, CRE significantly inhibited the mRNA expression of neurotrophins (BDNF, NGF, NT-3 and NT-4/5) in 12Z cells. Moreover, Akt overexpression markedly neutralized the inhibition of cell adhesion by CRE and expression of neurotrophins in 12Z cells. Furthermore, it was found that CRE suppressed NF-kB activation through the Akt pathway. These data suggest that CRE exerts anti-endometriotic activities by the inhibition of cell adhesion and neurotrophin expression, through the negative regulation of the Akt and NF-kB pathways in endometriotic cells.

## 1. Introduction

Endometriosis is a chronic gynecological disease causing chronic pelvic pain, dysmenorrhea, dyspareunia and infertility [1]. It is characterized by the presence and growth of endometrial tissue outside the uterus in the peritoneal cavity. It affects approximately 6–10% of women of reproductive age and 30–50% of women with chronic pelvic pain [2]. Although the exact etiology of endometriosis remains unclear, retrograde menstruation and implantation theories are widely accepted for endometriosis [2,3,4].

The adhesion of retrograde endometrial fragments onto the pelvic mesothelium is a key step in the initiation of endometriosis formation [5]. Several adhesion molecules, including P-cadherin (a predominant cadherin subtype) and matrix metalloproteinase-2 (MMP-2) are upregulated in ectopic endometrial lesions, and have been suggested to be critical factors in regulating the adhesion of ectopic endometrium to the peritoneal mesothelium and/or extracellular matrix (ECM) [6,7]. Recently, an endometriotic implant surrounded by an inflammatory environment has also been reported to be involved in the generation of endometriosis-associated pelvic pain, by releasing pro-nociceptive mediators [8,9]. Emerging studies have demonstrated that neurotrophins, as a family of nerve growth factors, including brain-derived neurotrophic factor (BDNF), nerve growth factor (NGF), neurotrophin-3 (NT-3) and neurotrophin-4/5 (NT-4/5), are observed in endometriotic lesions, resulting in pelvic pain in endometriosis via neurogenic inflammation [10,11].

*Cyperus rotundus* Linn. (Cyperaceae), also known as “nut grass” or “purple nut sedge” is a species of sedge native to Asia, southern and central Europe, and Africa, and it grows naturally in tropical, sub-tropical and temperate regions [12]. In Asia, the rhizome of *C. rotundus* (Cyperi rhizoma, CR) has been traditionally used as a herbal medicine for the treatment of stomach and bowel disorders, bacterial infection and inflammatory diseases [13]. In traditional Chinese medicine, CR is one of the oldest known medicinal plants used for the treatment of dysmenorrhea, a symptom of endometriosis [14]. Modern pharmacological studies have demonstrated that CR has several pharmacological and biological properties, including anti-inflammatory, anti-diabetic, anti-diarrheal, cytoprotective, anti-microbial, anti-bacterial, anti-oxidant, anti-pyretic, anti-analgesic and neuroprotective activities [15,16,17,18,19,20,21,22]. However, there is no scientific evidence demonstrating the effect of CR on endometriosis, a chronic gynecological disease characterized by the growth and adhesion of endometrial tissues outside the uterus, resulting in chronic and severe pelvic pain [23,24]. In this study, we aimed to investigate the effects of Cyperi rhizoma extract (CRE) on endometriosis, and to elucidate the underlying molecular mechanisms.

## 2. Materials and Methods

### 2.1. Plant Material and Preparation of the Extract

The rhizomes of *Cyperus rotundus* L. were obtained from a domestic Korean market (Kyungdong Crude Drugs Market, Seoul, Korea) in June 2011. The origin of the herbal material was identified by one of the authors (D.S. Jang), and a voucher specimen (CYRO1-2011) was deposited in the lab of Natural Product Medicine, College of Pharmacy, Kyung Hee University. The dried and milled plant material (2.8 kg) was extracted with 10 L of 80% ethanol (EtOH) three times by maceration. The extract was combined and concentrated in vacuo at 40 °C to give an 80% EtOH extract (CRE; 399 g). For in vitro experiments, CRE powder was dissolved in dimethyl sulfoxide (DMSO) (v/v) and filtered through a 0.22 μm disk filter.

### 2.2. Materials

Dulbecco’s modified Eagle’s medium (DMEM)/F12 medium, Medium 199 (M199), Opti-modified Eagle’s medium (Opti-MEM), fetal bovine serum (FBS), penicillin and streptomycin were obtained from Life Technologies Inc. (Grand Island, NY, USA). Phosphate buffered saline (PBS), DMSO, RNase A, leupeptin, aprotinin and phenylmethylsulfonylfluoride (PMSF) were purchased from Sigma-Aldrich Co. (St. Louis, MO, USA). 3-(4,5-Dimethylthiazol-2-yl)-2,5-diphenyl tetrazolium bromide (MTT) was purchased from Molecular Probes Inc. (Eugene, OR, USA). Antibodies against anti-phospho-Akt, anti-phospho-IkBα and anti-phospho-IKKα/β were purchased from Cell Signaling Technology (Beverly, MA, USA). The antibodies against anti-Akt, anti-p65, anti-IkBα, anti-IKKα/β, anti-PARP and anti-ß-actin were purchased from Santa Cruz Biotechnology (Santa Cruz, CA, USA). CellTracker^TM^ was obtained from Invitrogen (Grand Island, NY, USA).

### 2.3. Cell Culture

Immortalized human endometriotic epithelial cells (12Z) were established from active endometriotic lesions from women with endometriosis. In addition, the cell line displayed the in vivo properties of the active phase of endometriosis as well as the phenotypic characteristics [25]. The endometriotic 12Z cells were generously provided by Dr. Starzinski-Powitz (Johann-Wolfgang-Goethe-Universitaet, Frankfurt, Germany) and maintained in DMEM/F12 medium supplemented with 5% FBS, 100 U/mL penicillin and 100 mg/mL streptomycin (Life Technologies, Grand Island, NY, USA) in a humidified atmosphere of 5% CO_2_–95% air at 37 °C. The human mesothelial cell line Met5A were originally from the American Type Culture Collection. The cells were cultured in M199 supplemented with 10% FBS, penicillin (100 U/mL) and streptomycin sulfate (100 μg/mL).

### 2.4. Transfection

The pcDNA3-Akt-Myr (the constitutively active form of Akt) or the empty vector were obtained from Addgene (Cambridge, MA, USA). After cells were cultured to 60–70% confluence, the cells were transfected with plasmid DNA constructs (2 µg/mL) by polyethylenimine (PEI) in serum-free OPTI-MEM (Invitrogen, CA, USA), according to the manufacturer’s instructions. After 24 h, the medium containing the DNA plasmids was replaced with a fresh DMEM/F12 medium with 5% FBS, and the cells were treated with CRE.

### 2.5. Cell Viability Assay

Cell viability was assessed using an MTT assay. Briefly, human endometriotic cells (12Z) were seeded at a density of 9 × 10^4^ cells/mL in each well containing 50 μL of DMEM/F12 medium in a 96-well plate. After 24 h, various concentrations of CRE were added. After 24 h, 30 μL of MTT (1 mg/mL stock solution) was added, and the plates were incubated for an additional 4 h. The medium was discarded, and the formazan blue, which was formed in the cells, was dissolved in 50 μL DMSO. The optical density was measured at 540 nm using a microplate spectrophotometer (Spectra Max; Molecular Devices, Sunnyvale, CA, USA).

### 2.6. Adhesion Assay

The mesothelial Met5A cells were grown to confluence on 96-well plates. Endometriotic cells were detached by trypsinization, washed with phosphate-buffered saline (PBS), and probed with 10 µM CellTracker^TM^ for 45 min at 37 °C. CellTracker^TM^-labeled cells were washed with a DMEM/F12 medium containing 0.1% FBS to remove the free dye, and were added (2 × 10^4^ cells/well) to the mesothelial cells. After incubation at 37 °C for the indicated time, the non-adherent cells were removed by gentle washing, the fluorescence in each well was imaged, and the fluorescence was quantified in pixels using Scion Image Software (Scion Corp, Frederick, MD, USA).

### 2.7. Nuclear Extraction

Cells were washed with PBS, scraped into 1 mL of ice-cold PBS, and pelleted by centrifugation. The cell pellets were resuspended in hypotonic buffer (10 mM HEPES, pH 7.9, 1.5 mM MgCl_2_, 10 mM KCl, 0.2 mM PMSF, 0.5 mM DTT, 10 μg/mL aprotinin) and incubated on ice for 15 min. The cells were then lysed by adding 0.1% Nonidet P-40 and vortexed vigorously for 10 s. Nuclei were pelleted by centrifugation at 12,000× *g* for 1 min at 4 °C and resuspended in a high salt buffer (20 mM HEPES, pH 7.9, 25% glycerol, 400 mM KCl, 1.5 mM MgCl_2_, 0.2 mM EDTA, 0.5 mM DTT, 1 mM NaF, 1 mM Sodium orthovanadate).

### 2.8. Western Blot Analysis

Cells were washed with ice-cold PBS and extracted in a protein lysis buffer (Intron Biotechnology, Seoul, Korea). Protein concentrations were determined by the Bradford assay. The cell lysates were mixed with 5 Χ SDS sample buffer, boiled for 4 min, and then separated on 10% SDS-PAGE. After electrophoresis, proteins were transferred to polyvinylidene difluoride membranes. The membranes were blocked in 2% bovine serum albumin (BSA) for 30 min, washed, and incubated overnight at 4 °C with specific primary antibodies in Tris-buffered saline 2% BSA and 0.1% Tween-20 (TBS-T). The membranes were washed three times to remove the primary antibodies and incubated for 2 h with a horseradish peroxidase-conjugated secondary antibody (1:1000–2000). After washing three times with TBS-T, immuno-positive bands were visualized using the ECL chemiluminescent system (Amersham Pharmacia Biotech, ON, Canada) and analyzed using ImageQuant Las-4000 (GE Healthcare Life Science, WI, USA).

### 2.9. RNA Isolation and Real-Time RT–PCR Analysis

Total RNA was extracted from cells using the Easy Blue^®^ kits (Intron Biotechnology, Seoul, Korea), according to the manufacturer’s instructions. The total RNA was reverse transcribed into first-strand cDNA (Amersham Pharmacia Biotech, ON, Canada) following the manufacturer’s procedure. The synthesized cDNA was used as a template for polymerase chain reaction (PCR) amplification. Real-time PCR was performed using the Thermal cycler dice real-time PCR system (Takara, Japan). The primers used for SYBR Green real-time RT-PCR were purchased from Bioneer technology (Daejon, Korea) and are listed in Table S1. A dissociation curve analysis of P-cadherin, MMP-2, BDNF, NGF, NT-3, NT-4/5, and GAPDH showed a single peak. PCRs were carried out for 45 cycles using the following conditions: denaturation at 95 °C for 5 s, annealing at 57 °C for 10 s, and elongation at 72 °C for 20 s. The results for P-cadherin, MMP-2, BDNF, NGF, NT-3 and NT-4/5 mRNA were normalized to a control gene GAPDH.

### 2.10. Statistical Analysis

All the data were expressed as mean ± SD. The data obtained in experiments with multiple treatments were subjected to one-way ANOVA analysis of variance followed by a post hoc Tukey test of significance. Under all circumstances, *p* < 0.05 was considered to be significant.

## 3. Results

### 3.1. Cyperi Rhizoma Extract Inhibits Endometriotic Cell Adhesion to Mesothelial Cells

**Figure 1:**
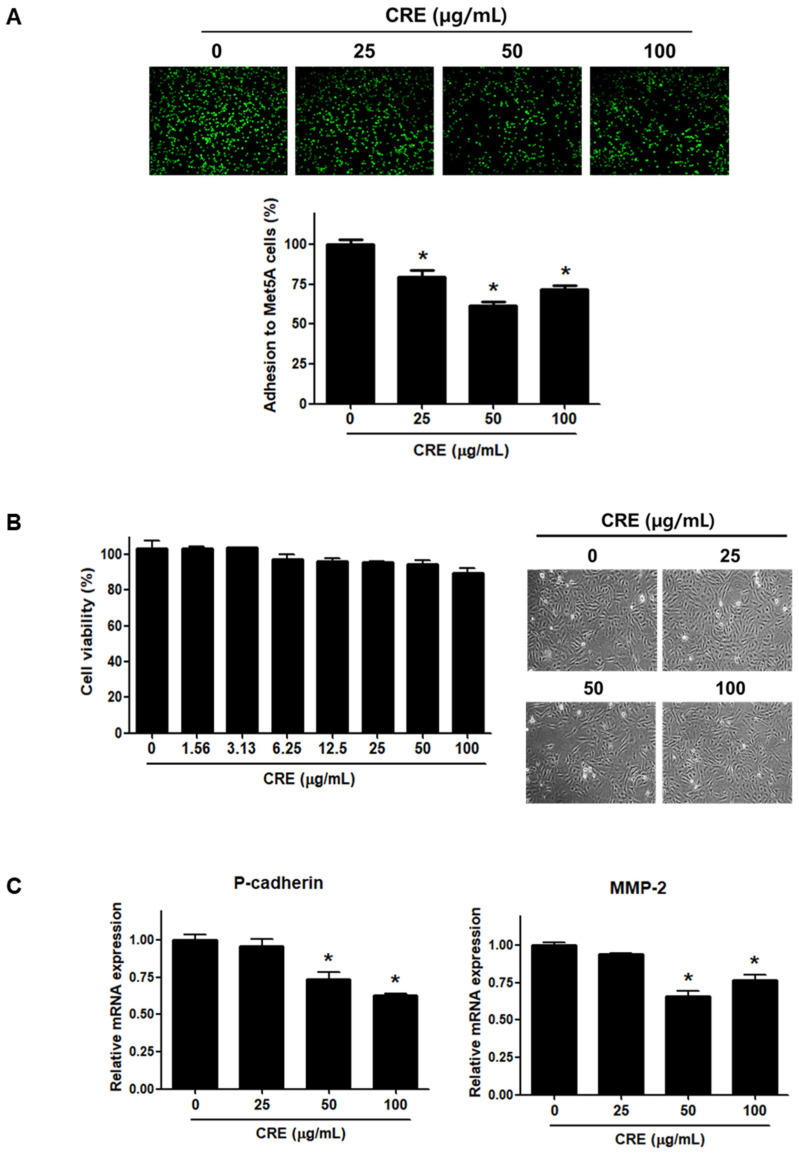
Effect of CRE on endometriotic cell adhesion to mesothelial cells and expression of adhesion molecules in endometriotic cells. (**A**) Human endometriotic cells (12Z) were treated with CRE at indicated concentration (25, 50 and 100 µg/mL) for 24 h. The 12Z cells labeled with CellTracker^TM^ (10 µM) were cultured on Met5A cell layers in a 96-well plate for 1 h. The total fluorescence in each well was measured by fluorescence microphotography. (**B**) Cell viability was determined by MTT assay. Representative images show the cell morphology of 12Z cells treated with CRE at indicated concentration (25, 50 and 100 µg/mL) for 24 h. (**C**) The mRNA expression of P-cadherin and MMP-2 was measured by real-time RT-PCR. All expression levels were normalized to GAPDH. Results are the combined data (mean ± SD) from three independent experiments. * *p* < 0.05 compared with control group.

Since the implantation of endometrial fragments at the peritoneum, which is covered by a mesothelial cell layer, is a key step in the establishment of endometriosis [24,26], we investigated the effect of CRE on the adhesion of endometriotic cells to mesothelial cells. As shown in Figure 1A, pre-treatment of human endometriotic 12Z cells with CRE (25, 50, and 100 µg/mL) significantly reduced the adhesion of 12Z cells to human peritoneal mesothelial Met5A cells. The inhibitory effect of CRE on the adhesion of 12Z cells to Met5A cells was not attributed to cytotoxic effects when the endometriotic cells were exposed to CRE up to 100 µg/mL for 24 h (Figure 1B). Furthermore, we investigated the expression of P-cadherin and MMP-2, which have been well known as potent determinants in peritoneal adhesion formation [5], after treatment with CRE in endometriotic 12Z cells. The results of real-time RT-PCR showed that CRE at concentrations of 50 and 100 µg/mL significantly decreased the mRNA expression of P-cadherin and MMP-2 in 12Z cells (Figure 1C).

### 3.2. Cyperi Rhizoma Extract Decreases the Expression of Neurotrophins in Human Endometriotic Cells

**Figure 2:**
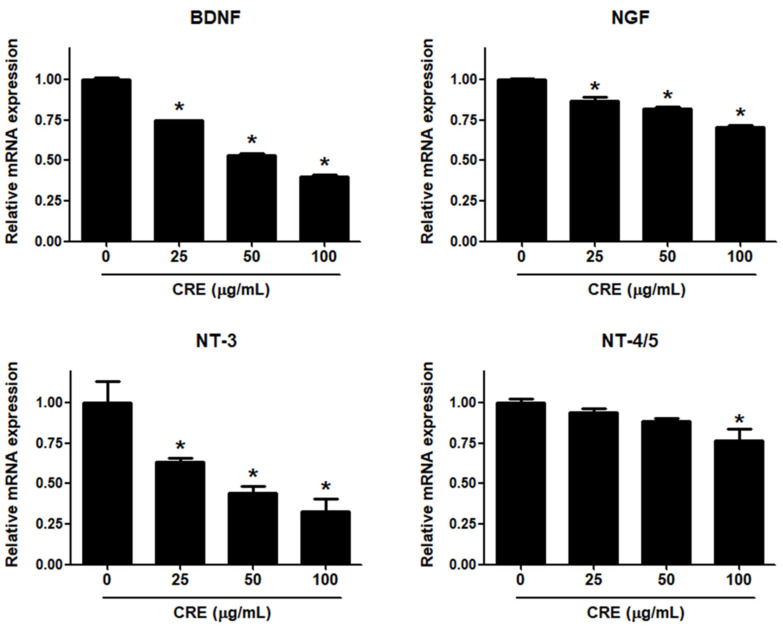
Effect of CRE on the expression of neurotrophins in endometriotic cells. The 12Z cells were treated with CRE (25, 50 and 100 µg/mL) for 24 h, and then the mRNA expression of neurotrophins (BDNF, NGF, NT-3 and NT-4/5) was measured by real-time RT-PCR. All expression levels were normalized to GAPDH. Results are the combined data (mean ± S.D.) from three independent experiments. * *p* < 0.05 compared with control group.

Neurotrophins, such as BDNF, NGF, NT-3 and NT-4/5, are overexpressed in endometriosis and are involved in the pathophysiology of pain generation in women with endometriosis [27]. In this regard, we investigated whether CRE has an inhibitory effect on the expression of neurotrophins in human endometriotic 12Z cells. When 12Z cells were treated with CRE, the mRNA expression of BDNF, NGF, NT-3 and NT-4/5 was inhibited in a dose-dependent manner (Figure 2). BDNF and NT-3 expression was markedly decreased by CRE at concentrations of 25, 50 and 100 µg/mL. NGF expression was slightly reduced by CRE stimulation at concentrations of 25, 50 and 100 µg/mL. NT-4/5 expression was significantly inhibited only at the highest concentration tested (100 µg/mL). Consequently, the highest dose (100 µg/mL) of CRE significantly inhibited the expression of all four tested neurotrophins in this study in endometriotic 12Z cells.

### 3.3. The Akt Pathway Is Involved in Cyperi Rhizoma Extract-Induced Anti-Endometriotic Effects in Human Endometriotic Cells

**Figure 3:**
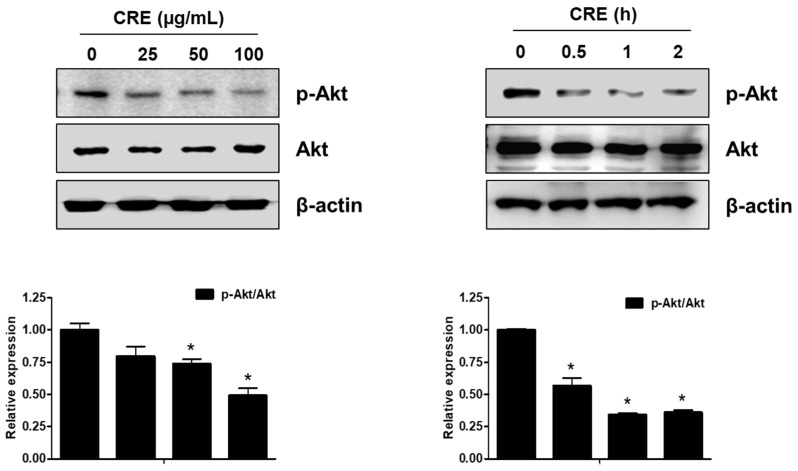
Effect of CRE on the phosphorylation of Akt in endometriotic cells. 12Z cells were treated with CRE (25, 50 and 100 µg/mL) for 2 h or with CRE (100 µg/mL) for 0.5, 1 and 2 h. Phosphorylated Akt and total Akt were measured by western blot analysis using specific antibodies. β-Actin was used as an internal control. Data are shown as mean band density. Data are presented as the means ± SD of three independent experiments; * *p* < 0.05 as compared with the control group.

**Figure 4:**
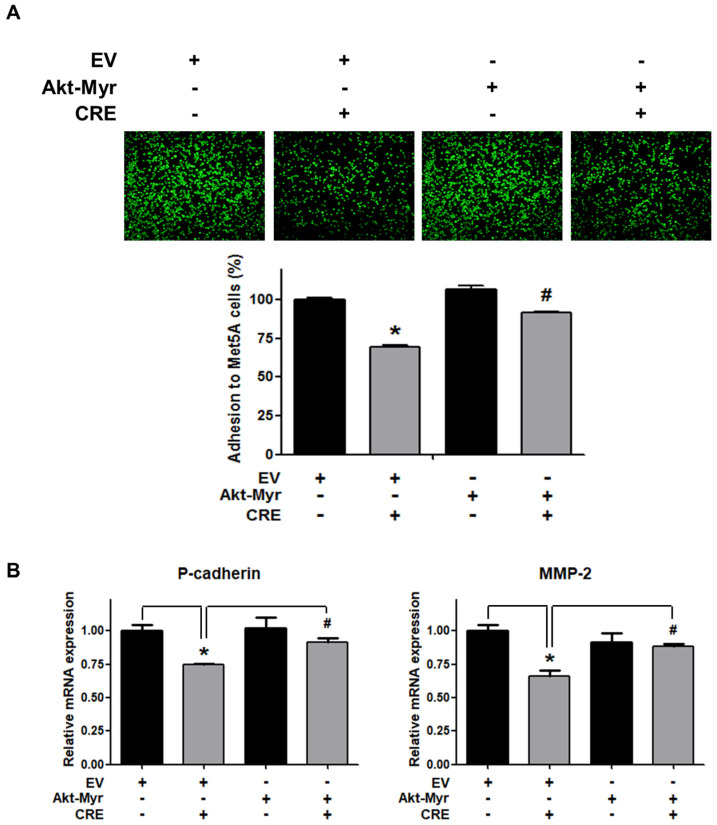
Involvement of the Akt pathway in endometriotic cell adhesion to mesothelial cells and expression of adhesion molecules in endometriotic cells. 12Z cells were transfected with an empty vector (EV) or a constitutive active form (Akt-Myr), and then the cells were treated with CRE (100 µg/mL). After 24 h, (**A**) an adhesion assay and (**B**) a real-time RT-PCR were performed. The mRNA expression of P-cadherin and MMP-2 was normalized to GAPDH. * *p* < 0.05 compared with EV-transfected none treatment group and ^#^ *p* < 0.05 compared with EV-transfected CRE treatment group.

**Figure 5:**
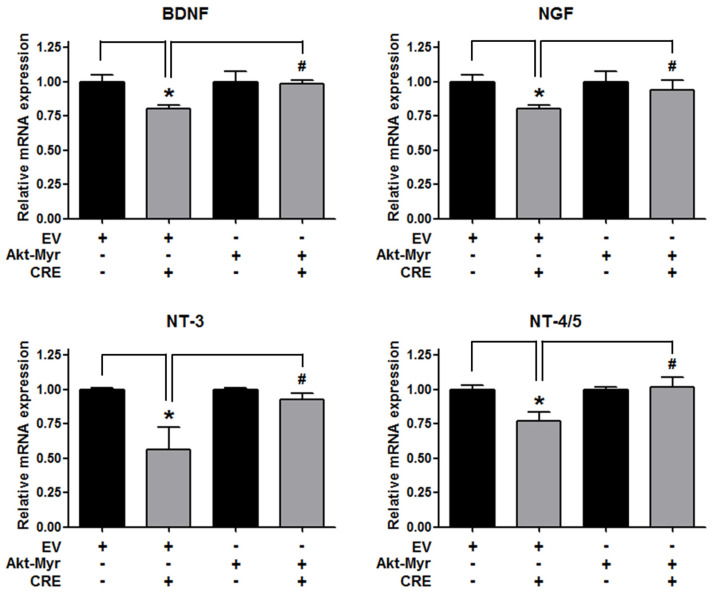
Involvement of the Akt pathway in the expression of neurotrophins in endometriotic cells. The 12Z cells were transfected with an empty vector (EV) or a constitutive active form (Akt-Myr), and then the cells were treated with CRE (100 µg/mL). After 24 h, real-time RT-PCR was performed to measure the mRNA expression of BDNF, NGF, NT-3 and NT-4/5. The expression levels were normalized to GAPDH. * *p* < 0.05 compared with EV-transfected none treatment group and ^#^ *p* < 0.05 compared with EV-transfected CRE treatment group.

The Akt pathway is a critical regulator of cell adhesion and migration through the regulation of actin organization and extracellular degradation [28]. Western blot analysis demonstrated that CRE significantly decreased Akt phosphorylation in 12Z cells at 50 and 100 µg/mL. In addition, CRE treatment inhibited the Akt activation in a time-dependent manner (Figure 3). To determine the involvement of the Akt pathway in the CRE-induced anti-endometriotic effect, we performed a cell adhesion assay and real-time RT-PCR analysis after transfection with a constitutively active form of Akt (Akt-Myr) in 12Z cells. As shown in Figure 4A,B, the overexpression of Akt partially reversed CRE-inhibited adhesion of 12Z cells to Met5A cells as well as the expression of P-cadherin and MMP-2 in 12Z cells, which was downregulated by CRE. In addition, the downregulation of neurotrophins by CRE was significantly attenuated by Akt overexpression in 12Z cells (Figure 5). These results suggested that CRE exerts anti-endometriotic effects through the downregulation of Akt activation in endometriotic cells.

### 3.4. Cyperi Rhizoma Extract Inhibits NF-kB Activation through the Akt Pathway

**Figure 6:**
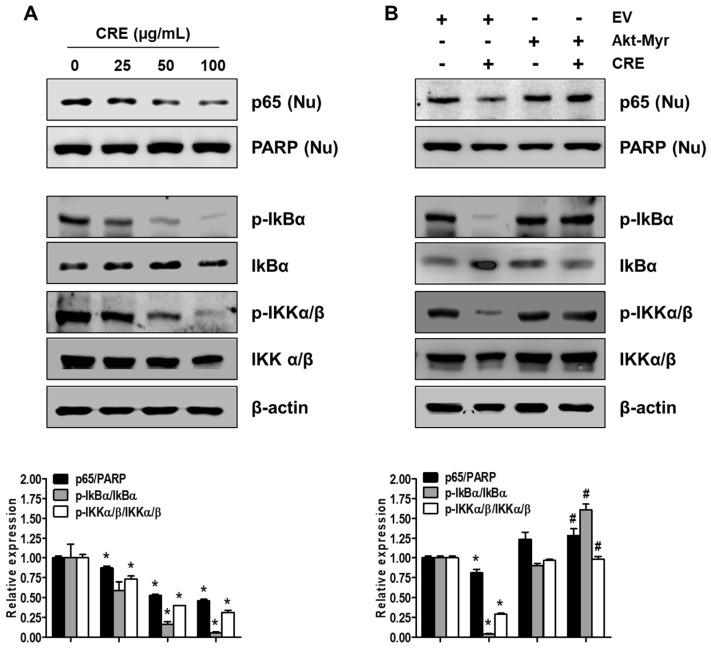
Effect of CRE on the NF-kB activation in endometriotic cells. Nuclear (Nu) and whole cellular proteins were isolated from the cells (**A**) treated with CRE (25, 50 and 100 µg/mL) for 8 h and (**B**) transfected with EV or Akt-Myr. The levels of p65 (Nu), p-IkBα, IkBα, p-IKKα/β and IKKα/β were detected by western blot analysis using specific antibodies. PARP and β-actin were used as an internal control for nuclear fractions and whole cell lysates, respectively. Data are shown as mean band density. Data are presented as the means ± SD of three independent experiments; * *p* < 0.05 as compared with the control group, and ^#^ *p* < 0.05 as compared with EV-transfected CRE treatment group.

Since NF-kB activation is implicated in the pathogenesis of endometriosis and an endometriosis-associated pro-inflammatory environment [29], we examined the effect of CRE on NF-kB activation in endometriotic 12Z cells. As shown in Figure 6A, CRE markedly reduced the nuclear translocation of p65 NF-kB in 12Z cells. In addition, the phosphorylation of both IkBα and IKKα/β, a key step in the modulation of NF-kB activation, was inhibited by CRE treatment in 12Z cells. To examine the involvement of Akt signaling in CRE-induced NF-kB inactivation in endometriotic cells further, we performed western blot analysis after transfection with Akt-Myr in 12Z cells. As shown in Figure 6B, NF-kB inactivation by CRE was reversed by Akt overexpression. In addition, Akt overexpression could recover the decrease in phosphorylation of IkBα and IKKα/β in the presence of CRE in 12Z cells. These results suggested that NF-kB inactivation by CRE is mediated by the Akt pathway in endometriotic 12Z cells.

## 4. Discussion

The rhizomes of *C. rotundus* have been traditionally considered one of the most important herbs for the treatment of obstetric/gynecologic disorders in Asia. In Vietnam and Taiwan, the decoction of *C. rotundus* is used for treating infertility and dysmenorrhea in women with endometriosis [14,30]. For example, Qi Gong Wan composed of various herbs including Cyperi rhizoma relieves mid-cycle pain, vaginal discharge and adhesions [14]. Although numerous experiences have been reported to show the effect of *C. rotundus* on gynecologic disorders [14,31], there is little scientific evidence of the effect of *C. rotundus* on gynecological diseases and the underlying molecular mechanisms of action. Choi et al. examined the effect of water-extracted tubers of *C. rotundus* on endometrial receptivity both in vitro and in vivo [32]. It was found that the water-extracted tubers of *C. rotundus* enhanced the adhesion of trophoblastic JAr cells to endometrial cancer Ishikawa cells for blastocyst implantation and improved the number of implantation sites in a mifepristone-induced implantation failure mice model. In a recent retrospective cohort study using the Taiwan National Health Insurance reimbursement database, the rate of endometriosis-related surgery, including hysterectomy and oophorectomy, was significantly lower in patients with endometriosis who took the traditional Chinese medicine formula Gui-zhi-fu-ling-wan, which mainly comprises *C. rotundus*, than in patients with endometriosis who did not take it [33]. Kim et al. has also demonstrated that Boheotang containing Cyperi rhizoma has an inhibitory effect on recurrent endometriosis after laparoscopic excision and hormone therapy [34]. However, to the best of our knowledge, there is no direct experimental evidence supporting the effect of *C. rotundus* on endometriosis. In this study, we investigated the effect of CRE on endometriosis using human endometriotic 12Z cells for the first time. The adhesive ability of endometriotic cells has been well observed in endometriotic lesion formation [26]. In this study, we demonstrated that CRE significantly inhibited the adhesion of endometriotic cells to peritoneal mesothelial cells, without cytotoxicity.

Previous phytochemical reports revealed the presence of flavonoids, tannins, alkaloids, glycosides, furochromones and sesquiterpenes in *C. rotundus* [35,36]. In fact, most of the biological activities of the rhizomes of *C. rotundus* have been attributed to the sesquiterpenes [36]. It is reported that isocyperol and α-cyperone, known as major sesquiterpene components of the rhizomes of *C. rotundus,* have various pharmacological activities, such as anti-inflammation, antivirulence, antigenotoxic, antibacterial and anti-depressant effects [37,38,39,40,41,42]. For example, isocyperol and α-cyperone have an inhibitory effect on inflammation through suppression of the NF-κB pathway [37,38]. Zhang et al. showed the inhibitory effects of α-cyperone on adhesion and invasion of avian pathogenic *Escherichia coli* O78 to chicken type Ⅱ pneumocytes [43]. However, to the best of our knowledge, there is no evidence supporting the effect of active constituents of CRE in gynecologic disorders including endometriosis. In a previous study, we found that α-cyperone is the most abundant and active component of CRE [44,45,46,47]. In these regards, we have examined the anti-endometriotic effect of α-cyperone in human endometriotic 12Z cells. Similar to CRE, α-cyperone significantly inhibited the adhesive ability of 12Z cells to mesothelial Met5A cells (Figure S1). This observation suggests a possibility that the anti-endometriotic effects of CRE are mainly attributed to its major component, α-cyperone. However, further experiments are still needed to draw this conclusion.

Cell surface proteins, including cadherin and MMPs, are expressed in the ectopic endometrium and implicated in the regulation of cell-cell adhesion as well as cell-ECM adhesion during the development of endometriosis [5]. Unlike E-cadherin and N-cadherin, P-cadherin is found to be remarkably increased in peritoneal endometriotic lesions and directly involved in the interaction between endometrial cells and peritoneal cells [6]. MMP-2 is a collagenase-degrading collagen IV, the principal component of basement membranes, and is significantly elevated in the urine of patients with endometriosis, compared to healthy women [48]. A recent study demonstrated that physiological changes, including adhesion, in endometriosis involve abnormal matrix remodeling, which is affected by proteolytic enzymes, such as MMP-2 [49]. In addition, MMPs support the irreversible inactivation of cadherin-catenin complexes [50]. In this study, we showed that CRE inhibited the mRNA expression of P-cadherin and MMP-2 in endometriotic 12Z cells. These results suggest that the downregulation of adhesion molecules, such as P-cadherin and MMP-2 by CRE, may contribute to the inhibitory effect of CRE on the adhesion of endometriotic cells to peritoneal mesothelial cells.

Pelvic pain is one of the major symptoms in women with endometriosis, and is associated with the activation of nociceptive neurons [51]. It has been suggested that the neurotrophins such as BDNF, NGF, NT-3 and NT-4/5 generate pelvic pain in endometriosis by the direct alteration of neurological responsiveness, leading to hyperalgesia and allodynia stimuli [1]. A recent study demonstrated that the level of BDNF in circulation is found to be elevated in women with endometriosis compared to healthy controls, and it decreased after the surgical removal of lesions [10]. In addition, it has been reported in a recent study that specific antibodies against neurotrophins, including NGF, may be used to treat and prevent pain and symptoms associated with endometriosis [52]. In the present study, we elucidated the inhibitory effect of CRE on the expression of BDNF, NGF, NT-3 and NT-4/5 in endometriotic cells. It was found that CRE significantly decreased the mRNA expression of BDNF, NGF, NT-3 and NT-4/5 in 12Z cells. Taken together, CRE may have an inhibitory effect on endometriosis-associated pain by modulating the expression of neurotrophins, which is elevated in women with endometriosis.

The PI3K/Akt pathway is well known to regulate various cellular events, such as cell growth, proliferation, apoptosis and differentiation stimulated by intra- and extracellular signals [53]. Recently, the role of PI3K/Akt signaling in endometriosis has been implicated. Yin et al. demonstrated that the over activation of the PI3K/Akt signaling pathway contributes to the decrease in decidualization of stromal cells from endometriosis [54]. In addition, rapid activation of PI3K/Akt mediated the growth factor- and estrogen-induced migration of endometrial stromal cells [55]. Moreover, it has been suggested that the activation of Akt signaling is involved in the expression of P-cadherin and MMP-2 [56,57]. In the present study, we found that the inhibitory effect of CRE on endometriotic cell adhesion and neurotrophin expression in endometriotic cells is mediated by the Akt pathway. Notably, it has been demonstrated that the activation of Akt affects the phosphorylation of IKKβ and the translocation of the NF-kB p65 subunit [58,59]. Many in vitro and in vivo evidences show that the constitutive activation of NF-kB in endometriotic lesions from patients with endometriosis promotes inflammation, cell proliferation, adhesion invasion and angiogenesis in an endometriosis-associated pro-inflammatory environment [60,61,62,63]. In these regards, we investigated the involvement of NF-kB activation in the effect of CRE on cell adhesion and neurotrophin expression in endometriotic cells through the Akt pathway. The results suggest that NF-kB signaling is likely to mediate the anti-endometriotic effect of CRE through the Akt pathway.

## 5. Conclusions

In summary, we demonstrated the inhibitory effect of CRE on endometriosis via the inhibition of cell adhesion and neurotrophin expression through negative regulation of Akt and NF-kB pathways in human endometriotic cells.

These results provide scientific evidence that CRE could be a candidate for multidisciplinary therapy, demonstrating a reduction of the adhesion of endometriotic fragments and pain relief in patients with endometriosis. The in vivo effect of CRE on endometriosis should be further investigated.

## Data Availability

Not applicable.

## Supplementary Materials

The following supporting information can be downloaded at: https://www.mdpi.com/article/10.3390/medicina58030335/s1, Figure S1: Effect of α-cyperone on endometriotic cell adhesion to mesothelial cells, Table S1: Primer sequences for real-time RT-PCR analysis, Supplementary Material and Methods: Preparation of the α-cyperone.
