# Supplementary material for: The Anti-Endometriotic Effect of Cyperi Rhizoma Extract, Inhibiting Cell Adhesion and the Expression of Pain-Related Factors through Akt and NF-kB Pathways
Source: Medicina (Kaunas). 2022 Feb 23;58(3):335. doi: 10.3390/medicina58030335 (PMC8953559; doi:10.3390/medicina58030335)
Supplement: Supplementary file 1 [file medicina-58-00335-s001.zip › medicina-1584701-supplementary.pdf]

Materials and Methods

Preparation of the α-cyperone

α-Cyperone was isolated from the Cyperi Rhizoma and identified by silica gel column chromatography, high-performance liquid chromatography (HPLC), and nuclear magnetic resonance (NMR), as described in previous studies [1-4]. The chemical structure of α-cyperone was identified by spectroscopic data and specific optical rotation and by comparison with published data.

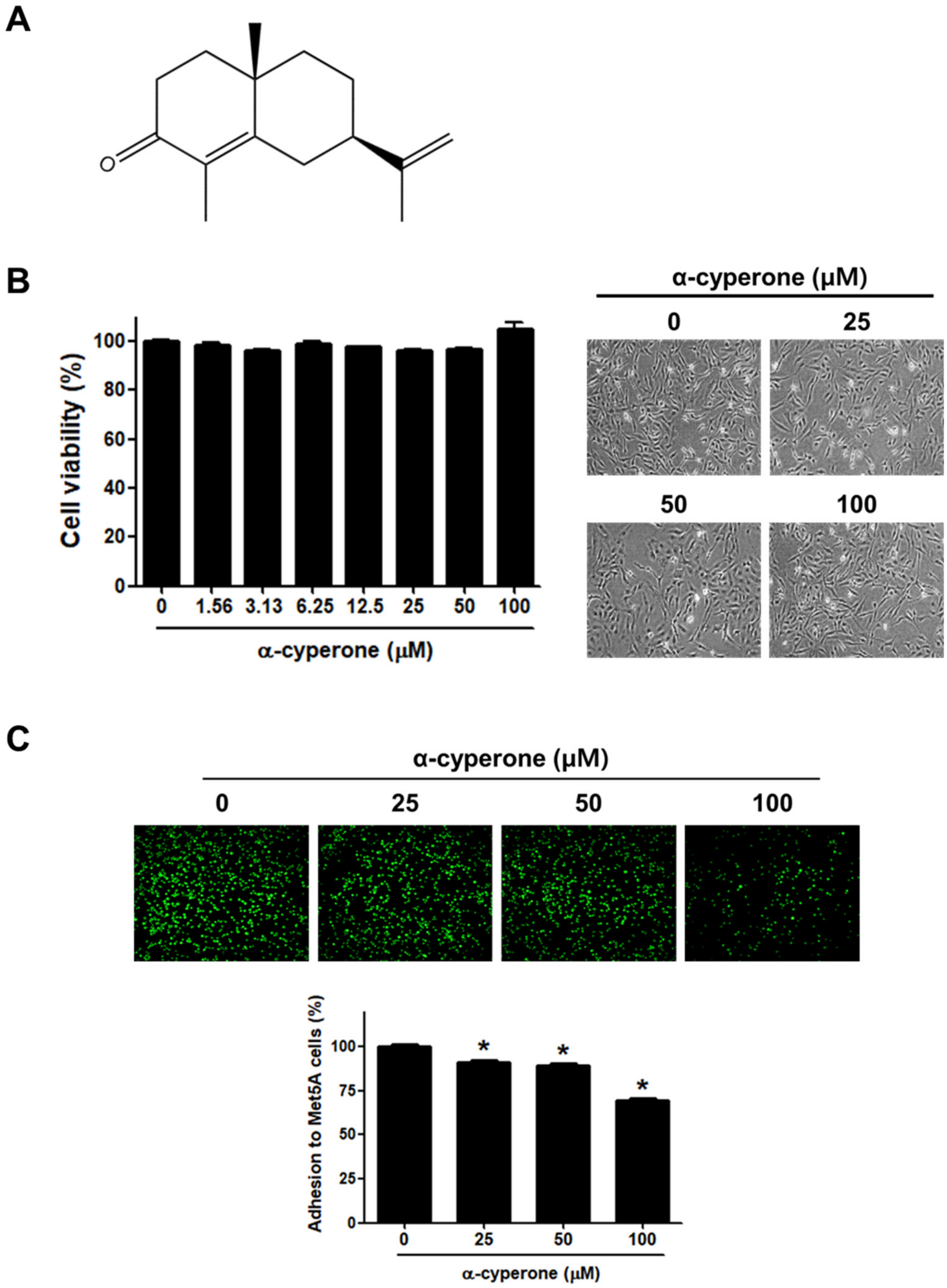

Figure S1. Effect of α-cyperone on endometriotic cell adhesion to mesothelial cells. (**A**) Structure of α-cyperone. The chemical structure of α-cyperone was identified by spectroscopic data and specific optical rotation and by comparison with published data. (**B**) Cell viability was determined by MTT assay. Representative images show the cell morphology of 12Z cells treated with α-cyperone at indicated concentration (25, 50, and 100 μM) for 24 h. (**C**) Human endometriotic cells (12Z) were treated with α-cyperone at indicated concentration (25, 50, and 100 μM) for 24 h. The 12Z cells labelled with CellTrackerTM (10 μM) were cultured on Met5A cell layers in 96-well plate for 1h. The total fluorescence in each well was measured by fluorescence microphotography. Results are the combined data (mean ± S.D.) from three independent experiments. *$P < 0.05$ compared with control group.

References

1. Kim, S.J., Kim, H.J., Kim, H.J., Jang, Y.P., Oh, M.S., Jang, D.S. New patchoulane-type sesquiterpenes from the rhizomes of *Cyperus rotundus*. *Bull Korean Chem Soc* **2012**, *33*, 3115-3118.
2. Kim, S.J., Ryu, B., Kim, H.-Y., Yang, Y.-I., Ham, J., Choi, J.-H., Jang, D.S. Sesquiterpenes from the rhizomes of *Cyperus rotundus* and their potential to inhibit LPS-induced nitric oxide production. *Bull Korean Chem Soc* **2013**, *34*, 2207-2210.
3. Ryu, B., Kim, H.M., Lee, J.-S., Cho, Y.J., Oh, M.S., Choi, J.-H., Jang, D.S. Sesquiterpenes from rhizomes of *Cyperus rotundus* with cytotoxic activities on human cancer cells in vitro. *Helvetica Chimica Acta* **2015**, *98*, 1372-1380.
4. Sim, Y.; Choi, J.G.; Gu, P.S.; Ryu, B.; Kim, J.H.; Kang, I.; Jang, D.S.; Oh, M.S. Identification of Neuroactive Constituents of the Ethyl Acetate Fraction from Cyperi Rhizoma Using Bioactivity-Guided Fractionation. *Biomolecules & therapeutics* **2016**, *24*, 438-445, doi:10.4062/biomolther.2016.091.
